# COVID-19-Associated Pulmonary Aspergillosis (CAPA) in Northern Greece during 2020–2022: A Comparative Study According to the Main Consensus Criteria and Definitions

**DOI:** 10.3390/jof9010081

**Published:** 2023-01-05

**Authors:** Panagiotis Siasios, Kostoula Arvaniti, Evangelia Zachrou, Aikaterini Poulopoulou, Pinelopi Pisanidou, Georgia Vasileiadou, Evangelos Kaimakamis, Athina Georgopoulou, Foteini Renta, Dimitrios Lathyris, Foteini Veroniki, Eleni Geka, Ioanna Soultati, Eleni Argiriadou, Eleni Apostolidou, Pinelopi Amoiridou, Konstantinos Ioannou, Leonidas Kouras, Ioanna Mimitou, Konstantinos Stokkos, Elliniki Flioni, Evangelos Pertsas, Maria Sileli, Christina Iasonidou, Evdokia Sourla, Georgia Pitsiou, Timoleon-Achilleas Vyzantiadis

**Affiliations:** 1 Department of Microbiology, Medical School, Aristotle University of Thessaloniki, 54124 Thessaloniki, Greece; 2 ICU, “Papageorgiou” General Hospital of Thessaloniki, 56403 Thessaloniki, Greece; 3 First ICU, “G. Papanikolaou” General Hospital of Thessaloniki, 57010 Thessaloniki, Greece; 4 ICU, “G. Gennimatas” General Hospital of Thessaloniki, 54635 Thessaloniki, Greece; 5 First ICU, “AHEPA” University General Hospital of Thessaloniki, 54636 Thessaloniki, Greece; 6 Second ICU, “AHEPA” University General Hospital of Thessaloniki, 54636 Thessaloniki, Greece; 7 ICU, “Bodossakio” General Hospital of Ptolemaida, 50200 Ptolemaida, Greece; 8 ICU, “Mamatsio” General Hospital of Kozani, 50100 Kozani, Greece; 9 ICU, “Agios Pavlos” General Hospital of Thessaloniki, 55134 Thessaloniki, Greece; 10 Second ICU, “G. Papanikolaou” General Hospital of Thessaloniki, 57010 Thessaloniki, Greece; 11 Respiratory Failure Unit, “G. Papanikolaou” General Hospital of Thessaloniki, 57010 Thessaloniki, Greece

**Keywords:** coronavirus disease 2019-associated pulmonary aspergillosis (CAPA), SARS-CoV-2 pandemic, diagnosis, invasive pulmonary aspergillosis, criteria, definitions, mycology laboratory

## Abstract

Coronavirus disease 2019 (COVID-19)-associated pulmonary aspergillosis (CAPA) has emerged as an important complication among patients with acute respiratory failure due to SARS-CoV-2 infection. Almost 2.5 years since the start of the COVID-19 pandemic, it continues to raise concerns as an extra factor that contributes to increased mortality, which is mostly because its diagnosis and management remain challenging. The present study utilises the cases of forty-three patients hospitalised between August 2020 and February 2022 whose information was gathered from ten ICUs and special care units based in northern Greece. The main aim was to describe the gained experience in diagnosing CAPA, according to the implementation of the main existing diagnostic consensus criteria and definitions, and present the different classification of the clinical cases due to the alternative algorithms.

## 1. Introduction

Invasive pulmonary aspergillosis (IPA) is a common complication in severely ill immunocompromised patients [1,2,3,4]. Over the past 2.5 years, the SARS-CoV-2 pandemic resulted in the admission of several patients to the ICU with severe clinical conditions, including COVID-19-associated pulmonary aspergillosis (CAPA), raising questions such as whether this superinfection could contribute to the increased mortality of said patients.

However, the incidence of CAPA varies widely not only within countries but even within hospitals in the same country, ranging between 3 and 33% [1]. The variability of CAPA reflects the difficulty in obtaining a reliable diagnosis, which is probably due to the lack of a specific clinical picture, the presence of non-specific radiological findings and the non-use of bronchoscopy, which, especially during the first waves of the pandemic, was limited due to the creation of aerosol and the risk of contamination of health care workers. Additionally, although serum galactomannan is a sensitive biomarker for patients with neutropenia, its sensitivity in non-neutropenic patients reaches only 25%, complicating its use in the diagnosis of CAPA [5,6].

Another important hint in diagnosing CAPA is the fact that the detection of *Aspergillus* species in specimens of the upper respiratory tract such as bronchial and tracheal secretions or sputum cannot differentiate between colonisation and infection [1,7,8].

Definitions of IPA were initially proposed in 2002 and updated in 2008 and 2019 by a consensus group of the European Organisation for Research and Treatment of Cancer and the Mycoses Study Group Education and Research Consortium (EORTC/MSGERC) [9,10], while the *Aspergillus* polymerase chain reaction (PCR) has been included as a microbiological criterion in 2019 [10]. The EORTC/MSGERC classification was only suitable for the immunocompromised patients who were not the majority of the ICU patients. 

In addition to the latter, the pandemic of the H1N1 virus in 2009 was the reason behind the development of the AspICU algorithm, which used clinical signs, less restrictive host factors and positive *Aspergillus* culture from the patient respiratory tract in order to define “putative” aspergillosis [11]. However, the AspICU algorithm did not use the galactomannan (GM) nor the *Aspergillus* DNA detection in blood samples or the broncho-alveolar lavage fluid (BAL) for the diagnosis of IPA. 

Additionally, both algorithms did not take into consideration the non-directed bronchoscopic lavage fluids (NBL) that were used in most of the cases for the diagnosis of IPA, especially in the first waves of the pandemic by SARS-CoV-2, when the protective equipment was limited. This fact resulted in the proposal of new diagnostic algorithms such as the novel CAPA definitions and the update of the current algorithms (modified AspICU algorithm) [11,12].

The present study included information from ten ICUs and special respiratory units from all around northern Greece (notably the region of Macedonia) according to the specimens’ referrals to the reference mycology laboratory of this rather large geographical area. 

During the SARS-CoV-2 pandemic in Greece, an annual increase (>50%) in respiratory samples that were referred to the aforementioned mycology laboratory for investigation of possible invasive fungal disease and an increase (131%) in positive cultures for *Aspergillus* species (Table 1) were observed. This increase followed the general trend of increase in COVID-19 cases in Greece [13]. The main purpose of the study was to describe the gained experience in diagnosing CAPA, according to the implementation of the existing diagnostic criteria and definitions and make it available for future analysis and possible modifications. In addition, we aim to present and discuss the different classification of clinical cases due to the use of alternative algorithms.

## 2. Materials and Methods

The Laboratory of Medical Mycology of the Medical School of the Aristotle University of Thessaloniki is a reference laboratory for fungal infections in northern Greece. The study included nine Intensive Care Units (ICUs) (seven from Thessaloniki, one from Kozani and one from Ptolemaida) and one unit of Intensive Respiratory Care (in Thessaloniki). Thessaloniki is the second largest Greek city and a main administrative centre for the whole geographical area. All participating units provided data on patients’ demographics, underlying medical conditions, risk host factors for invasive fungal infections and details on the diagnostic and therapeutic workup, including radiological data, treatment, and outcome of the patients via a questionnaire designed for the needs of the study.

Inclusion criteria of the study were the following:

- Adult patient (over 18 years of age) with SARS-CoV-2 infection confirmed molecularly by polymerase chain reaction (PCR).
- Admission to the ICU exclusively for the treatment of COVID-19 due to the concomitant respiratory failure. Patients with admission to the ICU due to other conditions, apart from the SARS-CoV-2 infection, were excluded.

The revised criteria of the European Organisation for Research and Treatment of Cancer/Mycoses Study Group (EORTC/MSG) [14], the consensus criteria for CAPA of the European Confederation of Medical Mycology and the International Society for Human and Animal Mycology (ECMM/ISHAM) [6], as well as the proposed clinical algorithm for the diagnosis of IPA in ICU patients (modified AspICU algorithm) [11,12] and the published “novel definitions” for CAPA [15] were all used for the classification of cases.

From the studied cases, none could be classified as a proven invasive pulmonary aspergillosis because a lung biopsy and/or an autopsy had not been performed on any of these patients.

From August 2020 until February 2022, forty-three cases of possible CAPA were identified; 26 male (median age 66.5 years, IQR 56.3–73.3) and 17 female (median age 58 years, IQR 42.0–66.0) patients. Mycology cultures were performed in 23 bronchoalveolar lavages and in 20 non-directed bronchoscopic lavages. Clinical laboratory workup of all patients included a complete blood count and serum biochemical tests, while the Sepsis-Related Organ Failure Assessment (SOFA) score at the time of admission at the ICU and at the time of respiratory sampling for mycological investigation were used to assess clinical severity. Ten patients (23.3%) were already vaccinated against coronavirus with one or more doses.

The study protocol was approved by the Bioethical Committee of the Medical School of the Aristotle University of Thessaloniki (ID number: 5.636/12.4.2022), and all patients’ medical data were anonymised.

An intensive diagnostic screening procedure for *Aspergillus* and/or other fungal species was implemented for all SARS-CoV-2 positive ICU patients and included:

1. Direct microscopy of the respiratory samples for the detection of fungal elements (hyphae, conidia, etc).
2. Mycology cultures by inoculation of the relevant respiratory samples on Sabouraud dextrose agar, malt extract agar and Czapek’s dox agar plates. All cultures were incubated at two temperatures (30 °C and 35 °C) for ten to twelve days in order to confirm the negative result.
3. Mycology cultures of peripheral and central venous catheter blood for investigating sepsis, with 5–10 mL of blood incubated up to 10–12 days and sub-cultured twice during this period.
4. Detection of galactomannan (GM) in respiratory samples and/or serum by the use of Platelia TM *Aspergillus* antigen assay (Bio-Rad, Marnes-la-Coquette, France) according to the manufacturer’s instructions. The cut-off for positivity was set at ≥0.5 for serum and ≥1.0 for bronchoalveolar lavage (BAL) or non-directed bronchoscopic lavage (NBL) specimens such as bronchial or tracheal secretions.
5. Implementation of qualitative in-house PCR for *Aspergillus* genus. Samples’ DNA was extracted according to the NucleoSpin^®^ Blood QuickPure method (Macherey-Nagel, Düren, Germany).
6. Detection of (1-3)-β-D-glucan (β-DG) in serum by the use of Fungitell^®^ assay (Associates of Cape Cod, E. Falmouth, MA, USA) or Dynamiker Fungus (1-3)-β-D-glucan assay (Dynamiker Biotechnology Co, Tianjin, China) following the manufacturers’ instructions, with a positivity threshold set at 80 pg/mL or 95 pg/mL, respectively.
7. Full identification (phenotypic and/or molecular) of the cultured fungi, either those grown on the inoculated samples in the mycology lab or those that were referred from the units of patients’ hospitalisation.
8. Antifungal susceptibility testing mainly by the use of appropriate strips of gradient antifungal concentration, Etest (bioMérieux SA, Marcy-l’Etoile, France) and MIC test strip (Liofilchem srl, Roseto degli Abruzzi, Italy).

All continuous variables were expressed as median (interquartile range, IQR), and univariate analysis was performed using Fisher’s exact and Wilcoxon tests, while a *p*-value of less than 0.05 was considered statistically significant. Kaplan–Meier survival curves and the log rank test were used for performing mortality analysis.

## 3. Results

Patients’ demographics and other characteristics of interest are described in Table 2. Briefly, 38 respiratory, 36 serum samples from 38 patients and 5 fungal culture plates of bronchial secretions from another 5 patients were referred to the mycology reference laboratory. Patients were mainly males (male/female sex ratio = 1.5) with a median age of 64 years (IQR, 53.5 to 70.5). Time duration of ICU stay ranged from 1 to 14 weeks with a median stay of 23 days (IQR, 19 to 37) and mostly implicated invasive mechanical ventilation (there was only one patient whose respiratory function was not supported mechanically). The time from molecular diagnosis of infection by SARS-CoV-2 until admission at the ICU was 11 days (7–15), while the time interval between ICU admission and sampling for mycological workup, which is the time of suspicion of CAPA, was 11 days (6–16.5). The mean time between ICU admission and CAPA diagnosis (receipt of final culture result) was 21 (17–26.5) days.

At the laboratory workup, lymphopenia (<1000 lymphocytes/μL) was found in 79% (34/43) of the patients admitted to the ICU. This laboratory finding seems to characterise patients with severe COVID-19 [16,17,18]. The lymphopenia continued at least until respiratory sampling for mycological investigation in most patients, 70.6% (24/34). An increase in inflammatory markers, CRP and procalcitonin (PCT), during the admission to the ICU and the relevant sampling, was found in 38.5% (15/39) and 38.1% (16/42) of patients, respectively. The deterioration of renal function with an increase in creatinine above normal levels was detected in 14% (6/43) of patients, while thrombocytopenia (<100.000 platelets/μL), either during admission to the ICU or at the time of respiratory sampling for mycological investigation, was found in 9.3% (4/43) and 20.9% (9/43) of patients, respectively.

Regarding the patients’ comorbidities (Table 3), the most frequent were arterial hypertension 46.5% (20/43), obesity 32.6% (14/43), diabetes mellitus and dyslipidaemia 30.2% (13/43). It is worth noting that among the above patients, seven (16.3%) presented risk factors according to the EORTC/MSG criteria (three chronic lymphocytic leukaemia, two multiple myeloma, one spinal ependymoma and one red blood cells dysplasia along with chronic corticosteroid use due to rheumatoid arthritis). One patient was pregnant at 32 weeks. Other underlying risk factors were Chronic Obstructive Pulmonary Disease-COPD (2/43) and being a regular (ex-) smoker (4/43).

Clinical symptoms suspicious for CAPA such as deterioration of respiratory function and increase in oxygen requirements were present in the majority of patients, 88.4% (38/43). Other clinical findings suspicious for CAPA included persistent respiratory failure for more than 5 days, despite the administration of appropriate treatment and ventilatory support 83.7% (36/43), tachypnoea 51.2% (22/43) and persistent fever and shortness of breath in 41.9% (18/43). Haemoptysis was present in 18.6% of the patients, while pleuritic rub was present in 9.3% (4/43).

Bilateral pulmonary infiltrates were found in all patients and were documented in 86% of patients by chest CT and in the remaining 14% by chest radiography. Imaging findings indicative of pulmonary aspergillosis such as nodules, caverns, Halo sign and sinusitis were found in 7%, 14%, 4.6% and 11.6% of patients, respectively.

Positive direct microscopic, i.e., hyphae characteristic for hyalohyphomycetes (comprising the *Aspergillus* genus) was present in 12 samples and more specifically in four out of the 23 BAL examined and in eight out of the 15 NBL. *Aspergillus* antigen detection was positive in 30 respiratory samples (15 BAL and 15 NBL) and in six serum samples. Serum galactomannan was positive in 23.1% (6/26) of CAPA cases. Positive culture of respiratory samples was found in 23 out of the 43 samples that came to the laboratory (7 BAL and 16 NBL). Microbiological details of culture, microscopy, PCR and GM testing are provided in Table 4 and Table 5.

Antifungal susceptibility testing was performed for 23 fungal strains (Table 5). In two patients, there was a mixed growth of filamentous fungi, which made impossible the antifungal susceptibility testing, and in one patient, it was decided not to perform the testing due to his earlier death.

Regarding pathogenic *Aspergillus* species in total, the most frequently isolated species in the cultures was *Aspergillus niger* species complex in 8/43 respiratory samples, either as pure growth (5/43) or in combination with other *Aspergillus* species (*Aspergillus fumigatus*: 1 culture) and *Candida albicans* (2 cultures), which was followed by *Aspergillus terreus* complex and *fumigatus* species complex, which were isolated in eight and seven respiratory samples, respectively.

More specifically, in non-directed bronchoscopic lavages, *Aspergillus niger* was the most frequently isolated *Aspergillus* species, while in bronchoalveolar lavages, it was *Aspergillus fumigatus*. In several cultures, more than one *Aspergillus* species as well as *Candida* species (9/43) were isolated. The results of the 43 respiratory specimens enrolled in the study are depicted in Table 6.

The non-growth of *Aspergillus* in the cultures of the respiratory specimens, despite the fact that there was a positive galactomannan in the respiratory specimen (BAL, NBL) or in serum or a positive PCR, could be attributed to the administration of voriconazole (early prophylactic antifungal treatment on suspicion of CAPA).

On the other hand, it is argued that heavy colonisation of the respiratory tract by *Candida* spp. may trigger galactomannan positivity [19]. In this study, three out of the fifteen positive BAL galactomannans were found in patients with positive cultures for *Candida albicans* (two cultures) and *Candida glabrata* (one culture), and two out of the fifteen positive NBL galactomannans were in patients with positive cultures for *Candida albicans* and *Candida parapsilosis*.

All patients received antifungal treatment for CAPA. Voriconazole and isavuconazole were the most frequent antifungal drugs used as monotherapy in 25 (58.1%) and 12 (27.9%) of patients, respectively, according to the recommended treatment for CAPA [7,20]. Of note, more than half of the patients received more than one antifungal drug, while in two patients, the type of antifungal treatment was not reported. The duration of antifungal therapy ranged from 2 to 98 days (median: 17 days).

Corticosteroids, in particular dexamethasone, were received by most of the patients as recommended by the international guidelines for patients in need of respiratory support [21]. The duration of corticosteroid therapy ranged from 5 to 61 days (median: 10 days).

In addition to the corticosteroids and antifungal agents, all patients received antimicrobial treatment during their hospitalisation in the ICU, mainly colistin, tigecycline, aminoglycosides and carbapenems, as well as antipseudomonal and antistaphylococcal penicillins. There were also four patients who received trimethoprim–sulfamethoxazole for prophylaxis against *Pneumocystis jirovecii*.

Finally, concerning the *Aspergillus* PCR in the respiratory specimens, it was performed in 15 out of the 43 patients and was found positive in 11 BAL out of 15.

### 3.1. Classification of Cases

All existing algorithms consist of several and sometimes different clinical, mycological and radiological criteria and are based even in different types of specimens or host factors. This fact could be the main cause of their different level of sensitivity in revealing CAPA.

The classification of patients according to the existing diagnostic criteria is presented in Table 7. According to the novel CAPA definitions, the requirements for putative CAPA are the existence of nonspecific radiology signs, associated with two or more positive results across different types of tests or multiple positives in one kind of test, such as positive culture from BAL/NBL, positive GM in BAL/NBL (≥1.0), positive PCR in blood or BAL/NBL, positive β-D-glucan in serum/plasma.

Using these definitions, all the CAPA cases of the study could be classified as putative. In 11 cases, there was only one positive mycological test, but the radiological findings were typical of IA, so they could be classified again as putative.

The above was the most sensitive classifier, which was followed by the ECMM/ISHAM consensus criteria (the most frequently used), which enabled the classification of 33 cases, out of 43, as CAPA (16 probable and 17 possible). The rest of the cases were considered as colonised. In the presence of clinical or radiological evidence, typical of IPA, a single positive bronchoalveolar lavage PCR result from the infected lobe is likely to be indicative of IPA [7]. This was the case in seven patients who had clinical symptoms, host factors and one positive PCR in bronchoalveolar lavage. However, according to the ECMM/ISHAM criteria, a confirmed diagnosis of CAPA requires two positive PCR results, so we considered these cases as *Aspergillus* colonisation, either permanent or transient. In three patients, the non-directed bronchoscopic lavage galactomannan index was less than 4.5, which is a prerequisite, according to the ECMM/ISHAM criteria, for the classification of cases as possible, and although the detection of galactomannan in non-directed bronchoscopic lavage is considered evidence for diagnosing CAPA [7], we also considered these three cases as cases of *Aspergillus* colonisation.

Using the modified AspICU algorithm, the incidence of CAPA in the study population was 46.5%. Twenty out of forty-three patients met the criteria for putative CAPA. Thirteen patients were considered as colonised.

An agreement between the ECMM/ISHAM and the modified AspICU algorithm was observed in 21 cases (20 CAPA and one *Aspergillus* colonisation), while 12 possible CAPA cases according to the ECMM/ISHAM criteria were classified as *Aspergillus* colonisation when examined with the modified AspICU algorithm. The modified AspICU algorithm was unable to classify 10 cases (nine with colonisation and one with possible CAPA according to the ECMM/ISHAM criteria).

The EORTC/MSGERC consensus criteria identified only seven cases of CAPA (three probable and four possible), in which patients had the needed host factors. For the rest of the cases, the aforementioned criteria were not applicable, as they did not have histopathological evidence or host factors (e.g., recent history of neutropenia, haematological malignancy etc.).

Concerning the classification of patients presenting host factors such as haematologic malignancy, prolonged use of corticosteroids, etc., the most sensitive classifier was the novel CAPA definitions by which all cases were considered as putative CAPA and the EORTC/MSGERC classification which classified three cases as probable CAPA and four cases as possible. The ECMM/ISHAM consensus criteria and the modified AspICU algorithm could not classify three cases, which was probably because PCR testing for *Aspergillus* was asked only once during the diagnostic investigation for each patient. Complete agreement between the EORTC/MSGERC and ECMM/ISHAM classifications was observed in three cases, while in one case, there was a downgrading of the severity of CAPA from probable to possible. Τhe sensitivity of ECMM/ISHAM and modified AspICU algorithms would be probably higher if more than one sample per each patient was referred to the Mycology Lab during the diagnostic investigation.

When classifying the cases according to the ECMM/ISHAM criteria (which are the more commonly used), the duration of antifungal treatment was longer in CAPA patients than in the colonised with *Aspergillus* (*p* = 0.029), while the time interval between molecular diagnosis of COVID-19 infection and admission to the ICU was shorter in the first group of patients (*p* = 0.00077). With regard to the mycological evidence for CAPA, galactomannan in BAL (depending on the analysis approach as qualitative, according to the negative or positive clinical evaluation of the result, or quantitative, according to the neat cut-off value) was higher in CAPA patients (*p* < 0.0001 and *p* = 0.0012, respectively) as well as the galactomannan in NBL fluids (*p* = 0.051). Levels of galactomannan in BAL and NBL fluids were always higher than those in serum. In addition, the cultures of NBL fluids were positive in the majority of CAPA patients, while in patients colonised with *Aspergillus*, they were negative (*p* = 0.0035). In total, the number of positive mycological criteria was bigger in the group of CAPA patients than in the colonised ones (*p* = 0.0011).

Host comorbidities, beyond the classical for IPA (severe immunosuppression, haematological diseases) such as arterial hypertension, mellitus diabetes, and obesity, have been described by many authors as the main risk factors for the development of invasive pulmonary aspergillosis in COVID-19 patients [22,23,24,25,26,27,28,29]. In this study, no association was found between the host comorbidities and the development of CAPA. Of note was the greater incidence of deep venous thrombosis (DVT) in the colonised patients (*p* = 0.0097).

### 3.2. Survival in Those with and without CAPA

**Figure 1:**
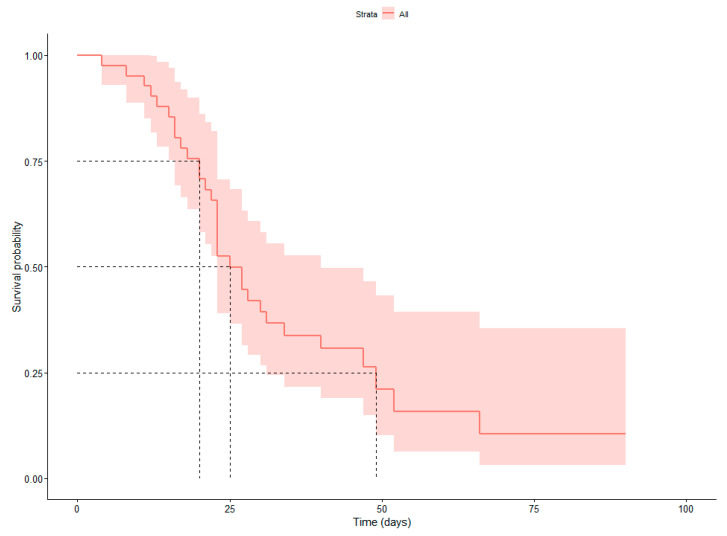
Survival probability curve for the patients admitted in the ICU due to COVID-19 infection.

Overall, 31 deaths were observed, while for two patients, no data for their survival was provided. Mortality was 24.2% versus 20.0% for CAPA or colonised patients by *Aspergillus* at their respiratory tract. It was estimated that half of the individuals admitted in the ICU lived longer than 25 days. Overall, 25% of patients died within 20 days after admission in the ICU, while the 75% of patients lived longer than 20 days. In contrast, 75% of individuals died within 49 days after their admission in the ICU (Figure 1).

**Figure 2:**
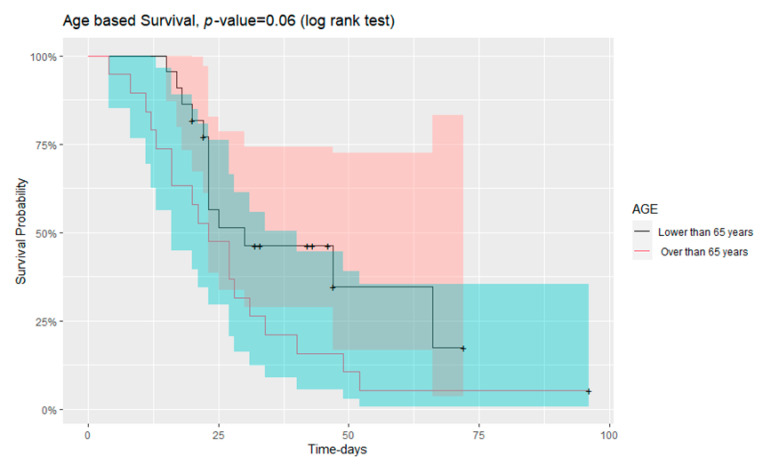
Kaplan–Meier survival curves for the mortality of patients enrolled, according to their age (log rank test, *p* = 0.06).

**Figure 3:**
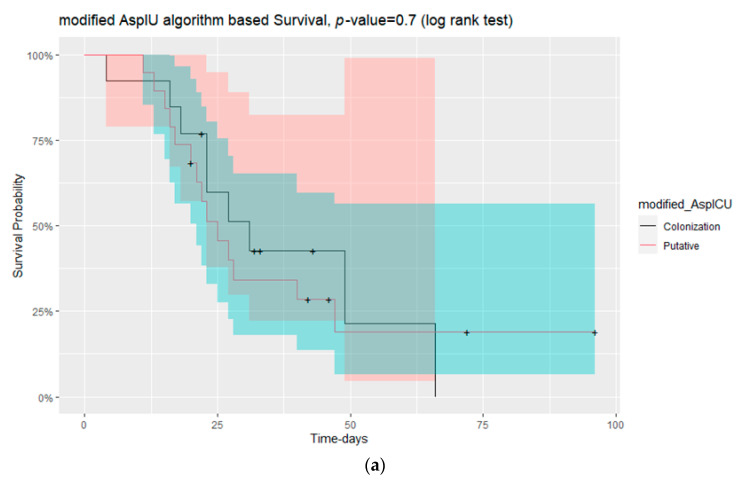

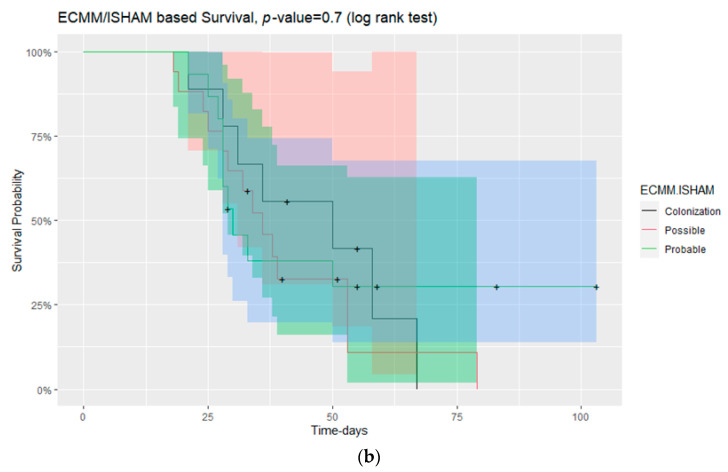
(**a**) Kaplan–Meier survival curves for the mortality of patients with putative CAPA or colonisation *by Aspergillus* according to the modified AspICU algorithm (log rank test, *p* = 0.7); (**b**) Kaplan–Meier survival curves for the mortality of patients with possible, probable CAPA or *Aspergillus* colonisation according to the ECMM/ISHAM criteria (log rank test, *p* = 0.7).

The mortality rate tended to increase in patients with age greater than 65 years old. The survival curve is shown in Figure 2 (long rank test, *p* = 0.06). Statistical analysis with Wilcoxon test also showed that the patients who died were older in age than those that survived (*p* = 0.035). There were no statistically significant differences in mortality rate according to sex between putative or *Aspergillus* colonisation patients (modified AspICU algorithm) and between possible, probable or *Aspergillus* colonisation patients (ECMM/ISHAM criteria) (Figure 3). On the contrary, mortality was significantly different in patients with thrombocytopenia either in admission in the ICU or during the time of respiratory sampling for mycological investigation (*p* = 0.04 and *p* = 0.05, respectively).

In addition, patients who passed away had a higher score of Sepsis-Related Organ Failure Assessment (SOFA) and counts of white blood cells or neutrophils during respiratory sampling in comparison to COVID-19 survivors (*p* = 0.017, *p* = 0.033 and *p* = 0.002, respectively). On the opposite, the stay in the ICU and the time of administration of voriconazole were longer in survivors than in patients who died (*p* = 0.007 and *p* = 0.025, respectively).

## 4. Discussion

The present study showed that the novel CAPA definitions were the most sensitive in comparison to other algorithms used for the classification of CAPA, being able to identify all cases and categorise them as putative IPA. The ECMM/ISHAM and the modified AspICU followed and were able to identify 33 (possible and probable) and 20 (putative) CAPA cases, respectively. The EORTC/MSGERC consensus criteria proved to be insufficient for CAPA definition in the general population of this study, as they rely mostly upon host factors and specific imaging signs. Therefore, even in other previous studies, they have misdiagnosed cases of IPA in the ICU [11,30]. The discrepancies in cases’ classification between the above algorithms demonstrate the fact that the identification between *Aspergillus* respiratory tract colonisation and “real” CAPA remains challenging.

The above are reinforced by the fact that although both categories (CAPA patients and patients colonised by *Aspergillus*) received antifungal treatment, as well as corticosteroids and antimicrobial treatment, as soon as possible, after their admission in the ICU, the mortality rates did not differ significantly between them.

Positive GM in NBL samples was detected in patients with colonisation (according to the ECCM/ISHAM criteria), while positive GM in BAL and in serum was detected only in patients with CAPA. The last supports the argument that although serum GM is not a highly sensitive biomarker, it is however highly indicative of IA [31]. It also shows the importance of bronchoscopy. A positive GM in BAL is more likely to be accompanied by an invasive disease [32].

The median time between ICU admission and CAPA diagnosis is estimated by several studies to be between 6 and 15 days [33,34,35]. In this study, the time interval between admission to the ICU and the diagnosis of CAPA by culture was 21 days (IQR: 18–26), while the time interval between admission to the ICU and the collection and referral of the respiratory specimen for mycological investigation (equals to clinical suspicion) was 11 days (IQR: 8–15). However, it should be emphasised that in several cases, the respiratory specimens were initially cultured in the hospitals in which patients were hospitalised and then referred to the mycology lab for confirmation of *Aspergillus* infection either as the initial samples or in combination with new specimens. Therefore, the real time of diagnosis was in most cases shorter than this mentioned above, and it is likely to be closer to the sampling day. It should be also mentioned that the galactomannan results of the respiratory and serum samples were reported to the ICUs at least 6–7 days earlier than the culture result and one to two days after the specimen arrival at the lab, providing an already serious suspicion for CAPA or even exclusion in many cases.

No association was found between the described patients’ comorbidities and the development of CAPA. The possible association between *Aspergillus* colonisation and DVT seems to be more attributable to the prevalence of the condition in the general population rather than a factor correlated to CAPA in ICU patients diagnosed with COVID-19.

An important conclusion, reinforced by other authors [36,37,38,39] as well, is the importance of the combined use of all possible available patient’s samples (bronchial secretions, tracheal aspirate) and methods to detect CAPA cases at the moment of the very first relevant sampling. Although non-cultured-based methods, such as PCR and/or GM, are not validated for respiratory specimens other than BAL, they are important in order to avoid under-diagnosis of CAPA, delayed initiation of therapy and subsequently increased patient morbidity and mortality.

The increment of respiratory samples that came to the laboratory, which matched the general increment of laboratory tests for mycological workup, indicates that the diagnostic strategy evolved towards more frequent respiratory diagnostic investigations during the pandemic.

A possible limitation of the study was that the majority of cases enrolled in this study did not have a referral of a second specimen in order to monitor their clinical situation and permit the extraction of more secure conclusions.

As it is also recommended by the ECMM/ISHAM consensus criteria, it is highly important, due to the severity and high mortality of CAPA, to collect prompt consecutive samples and refer them for specialised mycological investigation.

## Figures and Tables

**Table 1:** The annual increase in the numbers of respiratory samples and laboratory tests performed for fungal investigation in the Laboratory of Medical Mycology, Medical School, Aristotle University of Thessaloniki, during the studied period of the SARS-CoV-2 pandemic.

	Annual Increase (%) 2020–2021
Respiratory samples	52.5
Positive cultures for *Aspergillus* spp	131.3
Ag *Aspergillus*	32.5
PCR for *Aspergillus* genus	43.4

**Table 2:** Demographics and other characteristics of the patients enrolled in the study.

	Median (1st Qu–3rd Qu)			
Demographics	Total	CAPA Patients *	*Aspergillus* Colonisation *	*p*-Value
Males/Females	26/17	20/13	6/4	
Vaccinated against SARS-CoV-2	23.3% (10/43)	15.2% (5/33)	50% (5/10)	
Age (years)	64 (53.5–70.5)	64 (55–70)	64 (51.25–70.50)	0.86
Antifungal treatment- duration (days)	17 (10–30.5)	20 (12–35)	10 (7–18.75)	**0.029**
Stay in the ICU (days)	23 (19–37)	27 (20–42)	22.5 (14.75–28.25)	0.24
Days from diagnosis of COVID-19 infection by SARS-CoV-2 until admission at the ICU (days)	11 (7–15)	10 (6–14)	16.5 (13.5–21)	**0.0007**
Days of culture result after ICU admission (days)	21 (17–26.5)	21 (18–26)	20.5 (14.25–26.25)	0.45
Respiratory sampling time after ICU admission (days)	11 (6–16.5)	11 (8–15)	10.5 (4.25–17.25)	0.52
BMI	29 (26.4–31.55)	29.15 (26.82–31.77)	28 (25–31)	0.45
SOFA score (admission in ICU)	7 (7–8.75)	7 (7–8)	7 (6–9)	0.84
SOFA score (sampling)	8 (7–9)	8 (7–9)	8 (7–8)	0.88
corticosteroid use- duration (days)	10 (10–17.5)	10 (10–14)	10.5 (10–23.75)	0.58
WBC(a) (cells/μL)	13,400 (9425–18,510)	13,900 (10,300–17,600)	8435 (6258–20,675)	0.66
NEUT(a) (cells/μL)	11,422 (7965–16,625)	12,232 (8806–16,450)	6862 (5485–15,609)	0.20
LYMP(a) (cells/μL)	630 (471–930)	592.6 (491–880)	803.2 (446.9–1250.3)	0.28
PLT(a) (platelets/μL)	291,000 (172,500–343,500)	292,000 (212,000–322,000)	182,500 (126,500–355,250)	0.49
Hb(a) (mg/dL)	12.5 (10.7–13.2)	12.8 (11.9–13.2)	11.25 (8.95–13.10)	0.21
HCT(a) (%)	37.2 (32–40.1)	37.6 (34.8–40)	33.4 (27.75–40.02)	0.20
CRP(a) (mg/dL)	10.3 (6.3–16.45)	10.35 (5.73–16.3)	9.65 (7.28–15.98)	0.93
PCT(a) (μg/L)	0.195 (0.08–0.56)	0.2 (0.1–0.5)	0.2 (0.1–0.6)	0.62
Cr(a) (mg/dL)	0.7 (0.58–1.22)	0.7 (0.6–1.1)	0.7 (0.6–1.275)	0.98
WBC(s) (cells/μL)	11,530 (9135–16,555)	11,590 (8980–17,470)	10,420 (9708–12,848)	0.51
NEUT(s) (cells/μL)	9300 (7406–14,742)	9570 (7302–15,897)	8430 (7701–10,568)	0.19
LYMP(s) (cells/μL)	810 (520–1172)	810 (504–1070)	888.7 (570.6–1881.5)	0.55
PLT(s) (platelets/μL)	216,000 (132,750–279,750)	229,500 (145,500–277,250)	182,000 (101,500–378,250)	0.62
Hb(s) (mg/dL)	10 (8.65–11.25)	10.1 (8.7–11.4)	9.15 (7.98–10.7)	0.32
HCT(s) (%)	30 (26.95–34.25)	30.4 (27.1–34.4)	28.65 (26.93–31.8)	0.38
CRP(s) (mg/dL)	12.27 (4.93–22.05)	13.4 (4.98–22.45)	7.95 (4.78–15.93)	0.50
PCT(s) (μg/L)	0.33 (0.15–0.94)	0.3 (0.2–1.1)	0.4 (0.1–0.5)	0.54
Cr(s) (mg/dL)	0.75 (0.57–1.16)	0.8 (0.6–1.1)	0.8 (0.625–1.25)	0.42
VOR (treatment duration days)	8.95 (0–14)	6 (0–14.5)	2.5 (0–6.25)	0.51
GM BAL (Index)	2.19 (1.16–3.63)	1.945 (1.157–3.357)	0.14 (0.125–0.175)	**0.001**
GM NBL (Index)	3.9 (3.1–4.33)	4.075 (3.6–4.5)	1.3 (1.205–2.515)	0.051
GM serum (Index)	0.84 (0.6–1.06)	0.2 (0.09–0.4225)	0,09 (0.06–0.335)	0.24
Type of samples, n (%)				
BAL, n (%)	23 (53.5%)	16 (48.49%)	7 (70%)	
NBL, n (%)	15 (34.9%)	12 (36.36%)	3 (30%)	
Culture plates (NBL), n (%)	5 (11.6%)	5 (15.15%)	0	

CAPA: Coronavirus disease 2019-associated pulmonary aspergillosis, 1st Qu: First quartile, 3rd Qu: Third quartile, BMI: Body mass index, SOFA: Sepsis-Related Organ Failure Assessment, WBC: White blood cells, NEUT: Neutrophils, LYMP: Lymphocytes, PLT: Platelets, Hb: Hemoglobulin, HCT: Haematocrit, PCT: Procalcitonin, CRP: C-reactive protein, VOR: Voriconazole, GM: Galactomannan, BAL: Bronchoalveolar lavage, NBL: Non-directed broncoscopic lavage. (a): admission in ICU, (s): respiratory sampling. *p*-value ≤ 0.5 was considered as statistically significant and presented as bold, * classification of the cases as CAPA or colonisation by *Aspergillus* according to the ECMM/ISHAM consensus criteria.

**Table 3:** Comorbidities of the patients enrolled in the study.

Comorbidities	Total Patients n (%)	* CAPA Patients n (%)	* *Aspergillus* Colonisation Patients n (%)
**Cardiovascular system**			
Arterial hypertension	20/43 (46.5)	15/43 (34.9)	5/43 (11.6)
Atrial fibrillation	3/43 (7.0)	3/43 (7.0)	
Coronary angioplasty	1/43 (2.3)	1/43 (2.3)	
Coronary disease	5/43 (11.6)	4/43 (9.3)	1/43 (2.3)
Heart attack history	5/43 (11.6)	4/43 (9.3)	1/43 (2.3)
Heart failure	1/43 (2.3)	1/43 (2.3)	
Giant cell arthritis	1/43 (2.3)		1/43 (2.3)
Venous thrombosis	3/43 (7.0)		3/43 (7.0)
**Respiratory system**			
Bronchial asthma	3/43 (7.0)	2/43 (4.7)	1/43 (2.3)
Emphysema	1/43 (2.3)	1/43 (2.3)	
Obstructive sleep apnoea	2/43 (4.7)	1/43 (2.3)	1/43 (2.3)
Pulmonary embolism	1/43 (2.3)	1/43 (2.3)	
Chronic obstructive pulmonary disease	2/43 (4.7)	1/43 (2.3)	1/43 (2.3)
**Haematological/Ontological malignancies**			
Chronic lymphocytic leukaemia	3/43 (7.0)	1/43 (2.3)	2/43 (4.7)
Multiple myeloma	2/43 (4.7)	1/43 (2.3)	1/43 (2.3)
Malignancy	1/43 (2.3)	1/43 (2.3)	
Red blood cells dysplasia	1/43 (2.3)	1/43 (2.3)	
Spinal cord ependymoma	1/43 (2.3)	1/43 (2.3)	
**Kidney diseases**			
Chronic renal failure	1/43 (2.3)		1/43 (2.3)
**Disorders of endocrine glands**			
Diabetes mellitus	13/43 (30.2)	8/43 (18.6)	5/43 (11.6)
Hypothyroidism	4/43 (9.3)	2/43 (4.7)	2/43 (4.7)
Dyslipidaemia	13/43 (30.2)	12/43 (27.9)	1/43 (2.3)
**Other diseases**			
HBV carrier	4/43 (9.3)	3/43 (7.0)	1/43 (2.3)
Hyperuricemia	1/43 (2.3)	1/43 (2.3)	
Obesity	14/43 (32.6)	11/43 (25.6)	3/43 (7.0)
Osteoporosis	1/43 (2.3)	1/43 (2.3)	
Polymyalgia rheumatica	1/43 (2.3)	1/43 (2.3)	
Pregnancy	1/43 (2.3)	1/43 (2.3)	
Rheumatoid arthritis	1/43 (2.3)	1/43 (2.3)	
Beta thalassemia trait	1/43 (2.3)	1/43 (2.3)	
Ankylosing spondylitis	1/43 (2.3)		1/43 (2.3)
History of smoking or smoking	4/43 (9.3)	4/43 (9.3)	

CAPA: Coronavirus disease 2019-associated pulmonary aspergillosis, * classification of cases as CAPA or colonisation by *Aspergillus* according to the ECMM/ISHAM consensus criteria.

**Table 4:** Microbiological workup of the 43 patients enrolled in the study.

	CAPA Patients *	*Aspergillus* Respiratory Tract Colonisation Patients *	Total % (n)
Positive microscopic examination in BAL	4/16	0/7	17.4 (4/23)
Positive microscopic examination in NBL	8/12	0/3	53.3 (8/15)
Positive GM in BAL	15/16	0/7	65.2 (15/23)
Positive GM in NBL	12/12	3/3	100 (15/15)
Positive GM in serum	6/26	0/10	16.7 (6/36)
Positive culture for *Aspergillus* species in BAL	7/16	0/7	30.4 (7/23)
Positive culture for *Aspergillus* species in NBL	16/17	0/3	80 (16/20)
Positive PCR for *Aspergillus* genus	4/8	7/7	73.3 (11/15)

CAPA: Coronavirus disease 2019-associated pulmonary aspergillosis, GM: Galactomannan, BAL: Bronchoalveolar lavage, NBL: Non-directed broncoscopic lavage, PCR: Polymerase chain reaction, * classification of cases as CAPA or colonisation by *Aspergillus* according to the ECMM/ISHAM consensus criteria.

**Table 5:** Findings of microscopy, galactomannan, PCR and antifungal susceptibility testing in cases with positive culture.

	Sample	Species	Microscopy	GM			PCR	VOR MIC	IT MIC	CAS MIC	AP-B MIC	POS MIC	ISA MIC
				BAL	NBL	Serum							
1	BAL	*A. fumigatus*	Pos	4.16	-	0.36	-	0.25	0.75	0.125	0.047	0.19	0.19
2	BAL	*A. fumigatus A. niger*	Neg	4.6	-	0.54	-	0.094	0.38	0.19	1.0	0.094	0.19
								0.064	0.25	0.19	0.25	0.094	0.064
3	BS	*A. terreus and C. glabrata*	Pos	-	3.9	0.22	-	0.25	0.19	0.125	1.0	0.094	0.125
4	BAL	*A. fumigatus A. terreus*	Neg	2.52	-	0.05	Pos	0.094	1.0		<0.5		0.094
								0.023	0.016	0.094	8	0.094	0.012
5	BS	*A. fumigatus A. flavus*	Pos	-	4.15	-	-	0.47	0.75	0.19	1.0	0.125	0.094
								0.19	0.5	0.125	8	0.125	0.125
6	BAL	*A. fumigatus*	Neg	1.16	-	0.20	-	0.125	0.5	0.19	1.5	0.19	0.125
7	BAL	*A. terreus, A. flavus and C. albicans*	Neg	2.92	-	0.17	-	0.19	0.25	0.19	4	0.125	0.125
8	BS	*A. niger*	Pos	-	2.89	0.43	-	0.016	0.094	0.094	0.032	0.094	0.094
9	BS	*A. terreus*	Pos	-	5	0.4	-	0.125	0.19	0.094	1.5	0.094	0.094
10	BS	*A. niger*	Neg	-	4.5	0.08	-	0.032	0.094	0.094	0.125	0.094	0.094
11	CP (BS)	*A. flavus*	-	-	-	-	-	0.125	0.5	0.125	2.0	0.125	0.19
12	BS	*A. flavus*	Pos	-	3.7	0.2	-	0.19	0.38	0.047	4	0.094	0.094
13	CP (BS)	*A. niger*	-	-	-	-	-	Antifungal susceptibility test was not performed due to the patient’s death.					
14	BS	*A. flavus*	Neg	-	4.5	4.21	-	0.094	0.38	0.125	3	0.19	0.094
15	CP (BS)	*A. terreus*	-	-	-	-	-	0.125	0.38	0.094	6	0.094	0.19
16	BS	*A. niger and C. albicans*	Neg	-	4.05	0.22	-	0.25	2	0.25	1	0.38	0.75
17	BS	*A. niger and C. albicans*	Pos	-	4.7	0.03	-	0.19	1.5	0.19	0.125	0.125	0.25
18	BS	*A. niger*	Pos	-	3.3	0.67	-	0.25	2	0.19	1	0.5	0.5
19	CP (BS)	*A. terreus*	-	-	-	-	-	0.064	0.125	0.094	0.25	0.064	0.125
20	CP (BS)	*A. fumigatus*	-	-	-	-	-	0.047	1	0.032	0.016	0.064	0.064
21	BAL	*A. niger*	Pos	3.09	-	0.58	Pos	0.047	1	0.19	0.25	0.25	0.125

BAL: Bronchoalveolar lavage, NBL: Non-directed bronchoscopic lavage, BS: Bronchial secretions, CP: Culture plate, GM: Galactomannan, VOR: Voriconazole, IT: Itraconazole, CAS: Caspofungin, AP-B: Amphotericin-B, POS: Posaconazole, ISA: Isavuconazole, MIC: Minimal Inhibitory Concentration, Pos: Positive, Neg: Negative.

**Table 6:** Microorganisms isolated in respiratory samples.

Fungi Isolated in Respiratory Sample *	Total % (n)
*Aspergillus niger*	11.6 (5/43)
*Aspergillus terreus*	7.0 (3/43)
*Aspergillus flavus*	7.0 (3/43)
*Aspergillus fumigatus*	7.0 (3/43)
*Aspergillus fumigatus and flavus and terreus*	2.3 (1/43)
*Aspergillus fumigatus and niger*	2.3 (1/43)
*Aspergillus terreus and Candida glabrata*	2.3 (1/43)
*Aspergillus fumigatus and terreus*	2.3 (1/43)
*Aspergillus fumigatus and flavus*	2.3 (1/43)
*Aspergillus terreus and flavus and Candida albicans*	2.3 (1/43)
*Aspergillus niger and Candida albicans*	4.7 (2/43)
*Aspergillus terreus and Candida albicans*	2.3 (1/43)
*Candida parapsilosis*	2.3 (1/43)
*Candida albicans*	7.0 (3/43)
*Candida glabrata*	2.3 (1/43)
Negative	34.9 (15/43)

* The species names represent the species complex, ex. *Aspergillus niger species complex*.

**Table 7:** Classification of the study cases according to the existing criteria.

	EORTC/MSGERC	ECMM/ISHAM	Modified AspICU	Novel CAPA Definitions
Putative	-	-	20	43
Probable	3	16	-	-
Possible	4	17	-	-
Colonisation	-	10	13	-
NA (not applicable)	36	-	10	-

## Data Availability

The data presented in this study are available within the article.
